# Interventional angiography utilization for adult trauma patients in Trauma Centers across the United States: An observational study using the National Trauma Data Bank

**DOI:** 10.1097/MD.0000000000030900

**Published:** 2022-10-07

**Authors:** Ghassan Bou Saba, Romy Rahal, Rana Bachir, Mazen El Sayed

**Affiliations:** a Faculty of Medicine, American University of Beirut, Beirut, Lebanon; b Department of Emergency Medicine, American University of Beirut Medical Center, Beirut, Lebanon; c Emergency Medical Services and Pre-Hospital Care Program, American University of Beirut Medical Center, Beirut, Lebanon.

**Keywords:** embolization, emergency department, interventional angiography, outcome, trauma

## Abstract

Angiography and embolization are part of trauma management protocols for various injuries. This study examines the use of angiography and embolization use in trauma care across Trauma Centers in the United States.

We used the National Trauma Data Bank (NTDB) 2017 dataset in this retrospective observational study. Adult trauma patients (≥16 years) who underwent conventional angiography with or without embolization were included. A univariate analysis was carried out to describe patients’ demographic and injury characteristics as well as the time to angiography, angiography details, complications, and outcome (survival to hospital discharge: yes/no). One-year period prevalence proportion of angiography procedure was determined.

A total of 4242 patients were included. The 1-year period prevalence proportion of angiography procedure with or without embolization was 0.53% (95% confidence intervals: 0.527–0.529). The median age was 41 years (interquartile range: 27–58) with most patients being in the age group 16 to 64 (83.8%) and males (72.6%). Over half of the patients, 55.4% had an embolization procedure performed in addition to angiography. The mean time to angiography was 263.77 ± 750.19 minutes. The most common embolization sites were the pelvis (24.9%), spleen (11.8%), and liver (9%).

This study described angiography and embolization utilization in adult trauma patients in Trauma Centers in the US. Its findings provide the basis for future studies to examine more closely angiography/embolization utilization in specific subpopulations, and to create standardized risk stratification tools for trauma patients who are candidates for this procedure.

## 1. Introduction

Trauma is the leading cause of death in people younger than 40 years of age with exsanguination accounting for up to 1/3 of those deaths.^[1]^ Interventional radiology modalities to control hemorrhage started in the early 70s. Particularly, angiography and embolization are the most used interventional radiology (IR) procedures in trauma management.^[2]^ A standard angiogram involves the use of a catheter to introduce a dye into the arteries and visualize any leakage in the injured area via X-ray imaging indicating an active bleed. When compared to CT angiography, the use of the standard angiography is considered faster which is favorable in a critical trauma setting, has lower cost, and has direct therapeutic implications as the catheter is already in place and ready for intervention.^[2]^

A study by Pryor et. al exploring the evolution of angiography use between 2 time periods (1993–1995 and 2000–2002) in a level I trauma center in the US, showed a general decrease in the total number of angiograms done on trauma patients coupled by an increase in the proportion of therapeutic angiograms^[3]^ and multi-detector computed tomography (MDCT) use.^[3]^ For diagnostic evaluation, especially for multi-injury cases, MDCT has practically replaced angiography and use rate exceeded that of the Focused Assessment with Sonography for Trauma (FAST).^[2]^ Even though MDCT has high accuracy in detecting vascular injuries, the need for diagnostic angiography is still present when there is high clinical suspicion with negative CT findings.^[4]^ Moreover, angiography is the modality of choice for therapeutic intervention such as embolization, ensuring vascular patency or inserting intravascular devices^[2]^ with high clinical success rate of angioembolization for liver, spleen, renal, and pelvic injuries (79.8%, 88%, 90.92%, and 91.75% respectively).^[2]^

Most guidelines on the use of IR in the treatment of traumatic injury have been based on observational studies and case series, however to date there are no studies that describe IR utilization and characteristics of patients across trauma centers in United States. The objective of this study is to examine the use of angiography and embolization and to describe patients and injury characteristics in trauma care across US trauma centers using a large national database.

## 2. Methods

**Figure 1.**
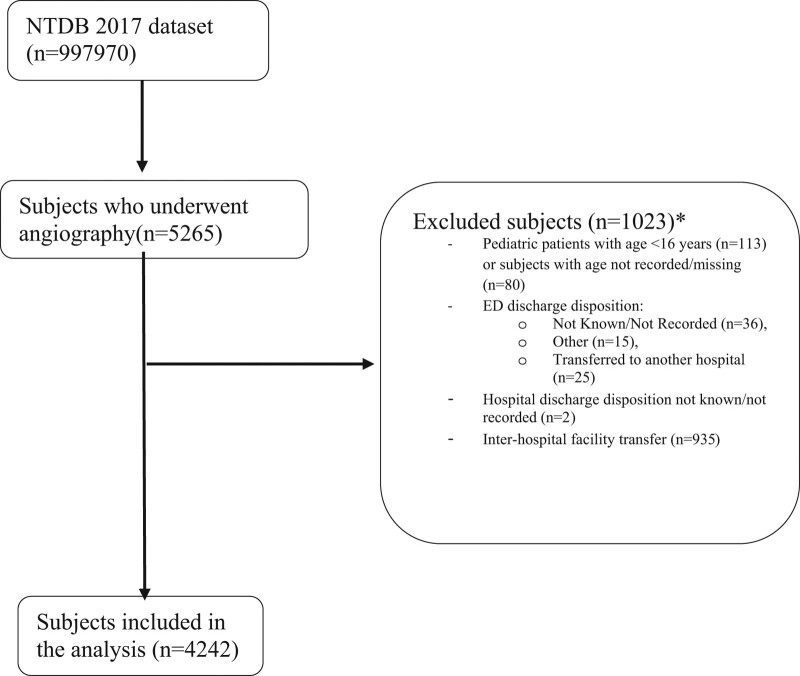
Study participants selection from the National Trauma Data Bank 2017. ED = emergency department. *There are overlaps among the categories of the excluded variables. More specifically, some patients who had inter-hospital facility transfer had as ED disposition one of the excluded categories. Also, some patients whose age was not recorded or were 15 years or younger were transferred or had as ED disposition one of the excluded categories. These overlaps explain why the final number on which the data analysis was conducted cannot be calculated just by subtracting the number of excluded patients from the selected sample.

This retrospective descriptive study used the National Trauma Data Bank (NTDB; American College of Surgeons; Chicago, Illinois USA) 2017 dataset (n = 997,970). NTDB is the largest trauma database in the US collecting information from more than 900 trauma centers with continuous quality checks. From patients who underwent angiography (n = 5265) we excluded patients whose age is ≤15 years (n = 113) which is considered the cutoff between pediatric and adult populations according to several trauma studies and to the NTDB criteria.^[5]^ Patients whose age was unknown (n = 80) were also removed from the sample as well as those who had emergency department (ED) discharge disposition recorded as “Not know/Not Recorded/Not Applicable” (n = 36), “other (jail, institutional care facility, mental health, etc)” (n = 15), “Transferred to another hospital” (n = 25), those with unknow hospital discharge disposition (n = 2) and those who had inter-hospital facility transfer (n = 935). The study population selection is presented in Figure 1.

No calculation was done for the required study sample as this is a descriptive study with no assessment of associations between variables, moreover, the data was extracted from the NTDB and as such it included all patients who met the inclusion criteria. Therefore, the occurrence of a selection bias is unlikely to happen because all eligible patients constituted the study sample.

Demographic data (age, sex, and race), clinical characteristics data (injury location, severity, mechanism of injury…), complications and outcomes information were collected and analyzed. We obtained an exemption letter from the Institutional Review Board at the American University of Beirut for the use of this is a de-identified data set. The datasets generated during and/or analyzed during the current study are available from the corresponding author on reasonable request.

### 2.1. Statistical analysis

We used the Statistical Package for the Social Sciences, version 24.0 (IBM, Armonk, New York, USA) to perform the statistical analyses. Categorical variables were described by calculating their frequencies and percentages, whereas continuous variables were summarized by computing their means (standard deviations), medians and interquartile ranges (IQR). No multiple imputation procedures were implemented to deal with the missing data because only descriptive analysis was conducted, and all variables had a missing value less than 5%. The 1-year period prevalence was reported as a percentage with the corresponding 95% confidence intervals (95% CI). The period prevalence was defined as being the proportion of patients who underwent an angiography procedure during a one-year period (numerator: N = 5265) divided by the total number of patients in NTDB 2017 (denominator: N = 997,970).

## 3. Results

The 1-year period prevalence proportion of angiography procedure with or without embolization was 0.53% (95% confidence intervals: 0.527–0.529).

A total of 4242 adult trauma patients were included in the full analysis with a median age of 41 years (interquartile range: 27–58). Most patients were in the age group 16 to 64 years (83.8%) and males (72.6%). In terms of race, 61.3% were white, 22.2% were African American and the rest were classified under “other race” or unknown/not recorded. The primary methods of payment were private/commercial insurance (40.5%), Medicaid/Medicare (35.1%) and self-pay (14.7%). Patients were mostly transported by ground ambulance (74.0%) to a level I trauma designated center (70.1%) (Table 1).

**Table 1:** General characteristics.

	Total
	N = 4242
**Age (years**)	
16–64	3556 (83.8%)
≥65	686 (16.2%)
**Sex**	
Male	3081 (72.6%)
Female	1161 (27.4%)
**Race**	
Black	942 (22.2%)
White	2601 (61.3%)
Other Race^*^	603 (14.2%)
Not known/not recorded	96 (2.3%)
**Ethnicity**	
Hispanic or Latino	627 (14.8%)
Not Hispanic or Latino	3427 (80.8%)
Not known/not recorded	188 (4.4%)
**Primary method of payment**	
Medicaid/medicare	1489 (35.1%)
Self-pay	625 (14.7%)
Private/commercial insurance	1717 (40.5%)
Not billed (for any reason) & other government & other	314 (7.4%)
Not known/not recorded	97 (2.3%)
**Transport mode**	
Ground ambulance	3138 (74.0%)
Helicopter ambulance & fixed-wing ambulance	900 (21.2%)
Private/public vehicle/walk-in	124 (2.9%)
Police & other	60 (1.4%)
Not known/not recorded	20 (0.5%)
**Facility level: hospital teaching status**	
Community	1304 (30.7%)
Non-teaching	392 (9.2%)
University	2544 (60.0%)
Not known/not recorded	2 (0%)
**Facility level: bed size**	
≤200	199 (4.7%)
201–400	962 (22.7%)
401–600	1223 (28.8%)
>600	1858 (43.8%)
**Trauma designation level**	
I	2973 (70.1%)
II	1208 (28.5%)
III	16 (0.4%)
Not known/not recorded	45 (1.1%)

* Other race is the combination of the following categories: Asian & Pacific Islander & American Indian & Other.

Most patients undergoing angiography had severe injury with an injury severity score (ISS) ≥ 16 (81.4%). All patients required blood transfusion within 4 hours of emergency department admission. Blunt trauma was most common (77.7%) followed by penetrating trauma (21.8%) with 77.1% of the overall injuries being unintentional, 17.2% being the result of assault, and 4% being self-inflicted. As for nature of injury, fractures (34.3%) and internal organ injury (38.2%) were most common with blood vessel injuries constituting only 9.8% of reported injuries (Table 2).

**Table 2:** Clinical characteristics.

	Total
	N = 4242
**Comorbidity**	
No	1860 (43.8%)
Yes	2382 (56.2%)
**ISS**	
≤15	788 (18.6%)
≥16	3451 (81.4%)
Not known/not recorded	3 (0.1%)
**GCS**	
Severe ≤ 8	1213 (28.6%)
Moderate 9–12	304 (7.2%)
Mild 13–15	2671 (63.0%)
Not known/not recorded	54 (1.3%)
**SBP**	
≤90	1235 (29.1%)
≥91	2911 (68.6%)
Not known/not recorded	96 (2.3%)
**Transfusion blood (4 h**)	
Yes	4242 (100%)
**Trauma type**	
Blunt	3297 (77.7%)
Penetrating	925 (21.8%)
Burn & other/unspecified	10 (0.2%)
Not known/not recorded	10 (0.2%)
**Injury intentionality**	
Unintentional	3270 (77.1%)
Self-inflicted	168 (4.0%)
Assault	729 (17.2%)
Undetermined & other	70 (1.7%)
Not known/not recorded	5 (0.1%)
**Mechanism of injury**	
Cut/pierce	229 (5.4%)
Fall	387 (9.1%)
Firearm	694 (16.4%)
MVT	2520 (59.4%)
Other^*^	344 (8.1%)
Not known/not recorded	68 (1.6%)
**Alcohol screen**	
No	1041 (24.5%)
Yes	3162 (74.5%)
Not known/not recorded	39 (0.9%)
**Drug screen**	
No	3119 (73.5%)
Yes	949 (22.4%)
Not known/not recorded	174 (4.1%)
**Nature of injury**	
Blood vessel	416 (9.8%)
Fracture	1454 (34.3%)
Internal organ injury	1622 (38.2%)
Open wound	331 (7.8%)
Superficial and contusion	215 (5.1%)
Other	195 (4.6%)
Not known/not recorded	9 (0.2%)
**Body region**	
Extremities	848 (20.0%)
Head and neck	1062 (25.0%)
Spine and back	335 (7.9%)
Torso	1949 (45.9%)
Unclassifiable by body region & unspecified	39 (0.9%)
Not known/not recorded	9 (0.2%)
**Signs of life**	
Arrived with no signs of life	37 (0.9%)
Arrived with signs of life	4205 (99.1%)

* Other mechanism of injury includes: fire/flame & machinery & pedal cyclist, other & pedestrian, other & transport, other & natural/environmental, bites and stings & natural/environmental, other & overexertion & struck by, against & other specified and classifiable & other specified, not elsewhere classifiable & unspecified.

ISS = injury severity score.

Over half of patients who underwent angiography (55.4%) had an embolization procedure performed as well (Table 3). Mean time to angiography was 263.77 ± 750.19 minutes. The embolization sites in decreasing order of frequency were pelvis (24.9%), spleen (11.8%), liver (9%), peripheral vascular sites (3.7%), kidneys (2.6%), aorta (2.4%), and retroperitoneum (1.4%). Hospital complications included unplanned intubation (4.6%), deep vein thrombosis (DVT) (5.5%), cardiac arrest with cardio-pulmonary resuscitation (CPR) (6.7%), pulmonary embolism (2.4%), unplanned return to the operating room (OR) (5.6%), extremity compartment syndrome (0.9%), and acute kidney injury (6.7%).

**Table 3:** Angiography/embolization procedure details.

	Total
	N = 4242
**Angiography procedure**	
Angiography only	1892 (44.6%)
Angiography with embolization	2350 (55.4%)
**Embolization site: liver**	
No	3862 (91.0%)
Yes	380 (9.0%)
**Embolization site: spleen**	
No	3742 (88.2%)
Yes	500 (11.8%)
**Embolization site: kidneys**	
No	4133 (97.4%)
Yes	109 (2.6%)
**Embolization site: pelvic (iliac, gluteal, obturator**)	
No	3185 (75.1%)
Yes	1057 (24.9%)
**Embolization site: retroperitoneum (lumbar, sacral**)	
No	4181 (98.6%)
Yes	61 (1.4%)
**Embolization site: peripheral vascular (neck, extremities**)	
No	4086 (96.3%)
Yes	156 (3.7%)
**Embolization site: aorta (thoracic or abdominal**)	
No	4142 (97.6%)
Yes	100 (2.4%)
**Embolization site: other**	
No	4036 (95.1%)
Yes	206 (4.9%)

In terms of outcomes, 45.5% of patients were transferred from the ED to the intensive care unit (ICU), 45.1% went to the OR, 7.6% transferred to regular units, telemetry step down units or observation units, 1.3% were discharged, and 0.6% died in the ED. Overall, 20% of patients died either in the ED or during hospital admission (Table 4).

**Table 4:** Clinical outcomes.

	Total
	N = 4242
**Hospital complication: central line-associated bloodstream infection (CLABSI**)	
No	4210 (99.2%)
Yes	32 (0.8%)
**Hospital complication: deep surgical site infection**	
No	4182 (98.6%)
Yes	60 (1.4%)
**Hospital complication: deep vein thrombosis (DVT**)	
No	4008 (94.5%)
Yes	234 (5.5%)
**Hospital complication: alcohol withdrawal syndrome**	
No	4194 (98.9%)
Yes	48 (1.1%)
**Hospital complication: cardiac arrest with CPR**	
No	3956 (93.3%)
Yes	286 (6.7%)
**Hospital complication: catheter-associated urinary tract infection (CAUTI**)	
No	4169 (98.3%)
Yes	73 (1.7%)
**Hospital complication: pulmonary embolism**	
No	4140 (97.6%)
Yes	102 (2.4%)
**Hospital complication: extremity compartment syndrome**	
No	4204 (99.1%)
Yes	38 (0.9%)
**Hospital complication: unplanned intubation**	
No	4048 (95.4%)
Yes	194 (4.6%)
**Hospital complication: acute kidney injury**	
No	3958 (93.3%)
Yes	284 (6.7%)
**Hospital complication: myocardial infarction**	
No	4221 (99.5%)
Yes	21 (0.5%)
**Hospital complication: organ/space surgical site infection**	
No	4198 (99.0%)
Yes	44 (1.0%)
**Hospital complication: osteomyelitis**	
No	4232 (99.8%)
Yes	10 (0.2%)
**Hospital complication: other**	
No	3782 (89.2%)
Yes	460 (10.8%)
**Hospital complication: acute respiratory distress syndrome (ARDS**)	
No	4079 (96.2%)
Yes	163 (3.8%)
**Hospital complication: unplanned return to the OR**	
No	4005 (94.4%)
Yes	237 (5.6%)
**Hospital complication: severe sepsis**	
No	4118 (97.1%)
Yes	124 (2.9%)
**Hospital complication: stroke/CVA**	
No	4153 (97.9%)
Yes	89 (2.1%)
**Hospital complication: superficial incisional surgical site infection**	
No	4193 (98.8%)
Yes	49 (1.2%)
**Hospital complication: pressure ulcer**	
No	4098 (96.6%)
Yes	144 (3.4%)
**Hospital complication: unplanned admission to the ICU**	
No	4074 (96.0%)
Yes	168 (4.0%)
**Hospital complication: ventilator-associated pneumonia (VAP**)	
No	4062 (95.8%)
Yes	180 (4.2%)
**ED and hospital dispositions**	
**ED discharge disposition**	N (%)
Floor bed (general admission, non-specialty unit bed)	223 (5.3%)
Observation unit (unit that provides <24 h stays)	8 (0.2%)
Telemetry/step-down unit (less acuity than ICU)	87 (2.1%)
Deceased/expired	26 (0.6%)
Operating room	1915 (45.1%)
Intensive care unit (ICU)	1929 (45.5%)
Home without services	54 (1.3%)
**Hospital discharge disposition**	
Deceased/expired	824 (19.4%)
Left against medical advice or discontinued care	30 (0.7%)
Discharged to home or self-care (routine discharge)	1104 (26.0%)
Transferred to other destination	2204 (52.0%)
Not applicable	80 (1.9%)
**Died ED/hospital**	
No	3392 (80.0%)
Yes	850 (20.0%)

CPR = cardio-pulmonary resuscitation, ED = emergency department, OR = operating room.

## 4. Discussion

This study describes the use of angiography and embolization in trauma care across Trauma Centers in the US using a national database. Variables examined in this population were general characteristics, clinical characteristics, time to angiography and angiography characteristics and outcomes including complications, and survival to hospital discharge. Previous similar studies were conducted on smaller scale samples and were restricted to organ-specific angiographies or to specific medical centers. To our knowledge, this is the first study to examine this topic of angiography in a comprehensive manner and at a national level. Its findings are important in clarifying practices in trauma care and in paving the road for more evidence-based protocols for angiography and embolization utilization.

The 1-year period prevalence proportion of angiography procedure with or without embolization was 0.53%. Patients had mostly severe injuries (81.4%), blunt trauma (77.7%) and the rate of angiography with embolization was high (55.4%) with pelvic embolization being the most common (24.9%). These findings were similar to those from previous smaller studies. One study that examined the use of angiography and embolization for abdominopelvic injuries in a Level I trauma center over the years from 1996 to 2010 showed a predominance of male patients and higher proportion of individuals identifying as white.^[6]^ The majority of patient in that study similar to ours had an ISS ≥ 16 (87% and 81.4% respectively), the most common mechanism of injury was motor vehicle accidents and the in-hospital mortality rate was nearly similar (16% vs 20% in our study).^[6]^ The same study also showed that patients who underwent angiography (n = 1300) had a higher injury severity score, higher mortality rates (16% vs 8% with no angiography) and longer length of hospital stay (21 days vs 10 days) than those who did not (n = 7845).^[6]^ Patients usually undergo angiography when there is evidence of occult bleeding, hypotension and severe injury requiring immediate blood transfusion.^[7,8]^ This patient population usually has high injury severity and require complex care with less favorable outcomes (mortality rate of 20%).

Most procedures were done at level I trauma centers (70.1%) and the reported hospital complications in this study were relatively low. Complications of embolization procedures are usually infarction of the solid organ that anatomically follows the embolized vessel, infection/abscess, and rebleed.^[3]^ In this study, the frequencies of infections were low: 0.8% of central line – associated bloodstream infection, 1.4% of deep surgical site infections, 1.0% organ/space surgical site infections, 1.2% superficial incisional surgical site infection, and 2.9% severe sepsis. On the other hand, 5.6% of patients had unplanned return to the OR and 4% had unplanned admissions to the ICU. These more common noninfectious complications could be related to a rebleed or to an infarction due to the angiography procedure or to other traumatic injuries. NTDB hospital complications variables are reported for the hospital course and are not directly attributed to a specific procedure such as angiography. A previous study in a level I trauma center looking at patients who had angiography between the years 2002 and 2008 (n = 97) showed that 29% of the patients had complications in the following 30 days after the procedure^[9]^: 24% were organ specific such as infarction, biloma, and necrosis, 3% were puncture site specific (hematoma), and 23% were systemic such as acute kidney injury and contrast induced nephropathy. The latter is common as most trauma patients are volume depleted and hypercoagulable. The low rate of overall complications can be related to the high volume of patients treated at level I trauma centers. Describing complications in terms of relation to procedures can be helpful in future NTDB datasets to allow for examining specific procedures’ outcomes.

Given the complexity of trauma injuries, modern trauma care is often managed through a multidisciplinary approach. During the past decade, the role of radiology in trauma management has fundamentally evolved.^[10]^ From being initially used as a diagnostic tool, it is now primarily adopted as a life-saving therapeutic tool in acute trauma settings.^[10]^ It is a low-risk and short-lasting intervention that is showing positive clinical outcomes in comparison to surgery.^[10,11]^ Therefore, assessing the use of IR in trauma care is important. This study should serve as a basis for future studies on different subsets of trauma patients. The findings will allow to establish clearer evidence-based guidelines for future practice. It is also important to explore factors associated with survival of trauma patients undergoing angiography in order to create risk stratification tools.

Potential limitations of this study are related to its retrospective nature and to the database used. While NTDB data is regularly monitored for variations in documentation and data is cleaned to ensure its validity, differences in reporting consistency might remain between the involved trauma centers. Additionally, some clinically significant variables were not taken into consideration as they are not accounted for or inconsistently reported in the NTDB such as prehospital interventions. Important secondary outcomes in trauma patients such as disability and functional status were also not included. These can be included among collected elements by NTDB to allow more detailed analysis of specific procedures such as angiography/embolization and corresponding outcomes. Despite these limitations, the NTDB is the largest trauma registry in the US and the findings of this study provide a national snapshot about the current use of angiography and embolization in US trauma centers.

## 5. Conclusion

In this study examining angiography and embolization in adult trauma patients, survival rate was relatively high 80.0% and hospital complication rates were relatively low. A detailed description of current practices related to IR procedures in trauma management was provided. The findings of this study provide the basis for future studies to examine more closely angiography/embolization utilization in specific subpopulations, and to create standardized risk stratification tools for trauma patients who are candidates for the procedure. This is in line with the trend to include interventional radiology suites near trauma bays for more timely interventions and improved outcomes.

## Author contributions

ME: Designed the study, oversaw the analysis and contributed to the writing and editing of the manuscript.

RB: performed the data analysis and contributed to the writing of the manuscript

GB and RR helped with the analysis tools and contributed to the writing of the manuscript
